# Emitter Location with Azimuth and Elevation Measurements Using a Single Aerial Platform for Electronic Support Missions

**DOI:** 10.3390/s21123946

**Published:** 2021-06-08

**Authors:** Mohamed Khalaf-Allah

**Affiliations:** Institute of Traffic Telematics, Technische Universität Dresden, 01062 Dresden, Germany; mohamed.khalaf_allah@tu-dresden.de

**Keywords:** emitter location, geolocation, three-dimensional (3D) positioning, angle of arrival (AoA), triangulation, azimuth, elevation, least squares (LS), particle filter (PF), electronic warfare (EW), electronic support (ES), unmanned aircraft system (UAS), radar warning receiver (RWR)

## Abstract

Passive ground emitter geolocation techniques are essential to electronic warfare systems, as they provide threat warnings in hostile environments, while ensuring the electronic silence of the mission platform. Geolocation of enemy emitters indicates the position of and type of adversary troops, and allows for the use of guided munition against enemy targets. Three-dimensional geolocation solutions based on least squares and particle filter estimation, using only azimuth and elevation measurements, were considered. Three batch-processing and one instantaneous solution algorithms, i.e., using a single pulse or a single observation point, were developed and investigated. The performance of the proposed solutions was demonstrated by simulations. Results showed that the batch-processing solutions achieved acceptable accuracies with a sufficient number of observation points. The performance degraded with fewer observation points. The instantaneous geolocation solution improved performance with increasing observation points, i.e., working in the sequential mode, and therefore could approach the accuracy of the batch-processing solutions.

## 1. Introduction

Passive localization (geolocation or location) of an emitter (or target) is an important issue in both civilian applications, such as government, scientific, and commercial, and in military missions. Some examples of applications include searching for emergency beacons, tracking radiofrequency (RF)-tagged wildlife, and finding unauthorized or adversaries’ radio sources [1]. The locating (or receiving) platforms measure the electromagnetic (EM) emissions of a target, thus no emission of energy is required. The extracted signals are then processed and analyzed to estimate the target’s location. This procedure is usually referred to as an emitter location or localization, an emitter geolocation, if the emitter is on the surface of the earth, or a direction finding (DF), if only angle, i.e., azimuth and elevation, measurements are involved. Location, localization, or geolocation are general terms for position estimation involving angle, range, or a combination of these measurements. DF, as the name suggests, involves only angle measurements. However, geolocation and DF are sometimes used interchangeably in the literature, since the early geolocation systems used angle-based methods. The EM environment is very important to military operations. Electronic warfare (EW), which is one of the core capabilities in military missions [2], covers a broad mix of military tactics, techniques, procedures (TTP), technology, and organizational structures that safeguard the use of the EM spectrum by friendly forces, denying its use by enemies, and defeating enemies’ efforts to achieve the same objectives [3]. The development and application of EW concepts and technologies continue to remain a high priority since EW is a powerful force multiplier. Therefore, many aspects of EW stay highly classified. However, EW is related to many civilian applications and technologies, e.g., spectrum monitoring and radio astronomy.

The roles of EW usually consist of three major branches (or divisions): electronic support (ES), electronic attack (EA), and electronic protection (EP). The main objective of ES, previously known as electronic support measures (ESM), is the sensing of radar, communication, and other EM signals of potential interest, emitted by adversary sources, for operational advantages. EA, previously known as electronic countermeasures (ECM), concerns active or passive efforts to degrade the enemy’s ability to use the EM spectrum. EP, previously known as electronic counter-countermeasures (ECCM), involves technologies and techniques to safeguard the electronic systems of friendly forces for effective operation in hostile EM environments, i.e., enemy ES and EA activities [3].

Measurement approaches for passive localization are based on time, angle, phase, frequency, amplitude, spatial information, or a combination of these. Common techniques include the time of arrival (ToA), the time difference of arrival (TDoA), the angle of arrival (AoA), the phase difference of arrival (PDoA), the frequency difference of arrival (FDoA), also known as differential Doppler (DD), the received signal strength (RSS), and the power difference of arrival (PDoA), also known as received signal strength difference (RSSD), of the emitted signals [4,5,6,7,8,9,10]. Please note the ambiguous PDoA acronym, where P may stand for phase or power. In the ToA, TDoA, AoA, PDoA, RSS, and RSSD schemes, the receivers and emitter might be stationary or moving, while the FDoA scheme requires relative motion between the receivers and emitter to generate the frequency difference necessary for the measurements. The frequency difference can be produced if either the receivers or emitter are moving, or if the receivers and emitter are moving at different velocities.

From a single platform, AoA measurements are often the best that can be obtained for geolocation of adversary signal sources, particularly if the signal emitter is sufficiently slow relative to the receiving platform [9]. Precision geolocation is enabled by the utilization of multiple platforms and stations to obtain TDoA or FDoA measurements. Hybrid TDoA and FDoA systems enable a single pair of receivers, under certain restrictions, to perform emitter geolocation [9]. FDoA systems are not widely considered as TDoA systems, due to issues of generating sufficiently accurate frequency measurements and their additional complexity. FDoA measurements are often considered in combination with TDoA measurements [5], and not in isolation, mainly to reduce the number of required platforms and because FDoA and TDoA measurement errors are often complementary [9]. A minimum of two platforms are required for emitter location in 3D space when using only FDoA measurements, provided the emitter is stationary and active for a longer observation period to allow the platforms to transmit a larger baseline [9].

Error sources that impact localization methods include propagation effects, e.g., multipath and non-line of sight (NLoS) signal propagation, environmental noise, interfering signals, receiver thermal noise, gain and phase mismatches in the cables and receivers in multi-receiver systems, mutual coupling between pairs of elements in antenna arrays, geometric factors due to the relative position of the emitter and receivers, and uncertainties in the position of the receivers. Increasing the number of receivers and optimizing the emitter-receiver geometrical layout contribute to the minimization of errors.

Small-size, low-cost, autonomous unmanned aircraft systems (UASs) can be a major support to a squad of soldiers, a small group of first responders, or emerging commercial interests [1]. The passive location of emitters is an important function in defense suppression and weapon delivery systems. The estimation process is accomplished by a single moving platform collecting successive measurements over time, or by multiple platforms collecting simultaneous measurements [11]. Instantaneous emitter location using a single pulse, i.e., a monopulse, from a single platform is an interesting feature of ES systems. Instant three-dimensional (3D) geolocation of a large number of emitters, spread over wide areas, is anticipated to become standard in airborne intelligence, surveillance, and reconnaissance (ISR) missions [12]. Parameters that can be measured using a single pulse, i.e., monopulse parameters, include azimuth and elevation AoA and ToA measurements.

The two-dimensional (2D) emitter location problem, using azimuth measurements, was solved by a closed-form (CF) small error approximation of the maximum likelihood (ML) estimation [13], which was further developed in [14,15] by iterative approaches. These approaches used Taylor series expansion around an initial estimate to use the linearized least squares (LS) estimation, iterative nonlinear least squares (NLS) estimation [16], ML estimation [17], CF linear least squares (LLS) estimation [18,19], which was improved in [20] by weighted least squares (WLS), CF total least squares (TLS) estimation [21], CF constrained total least squares (CTLS) [22], the discrete probability density (DPD) method [23], and the polynomial chaos expansion (PCE) theory [24]. The 3D emitter location problem, using azimuth and elevation measurements, was solved iteratively in [25], and by a CF algorithm in [26], which was an extension of the solution in [18] to 3D space. Weighted algorithms require a priori knowledge about the distribution of measurement errors, which may not be available in practical applications. Iterative approaches require a good initial guess to ensure convergence on the true position. Moreover, the iteration procedure can be computationally intensive. Therefore, CF solutions are attractive due to their small computational requirements, good performance, and their capacity to initialize iterative algorithms if they are still needed.

The approach in this article focuses on passive techniques to locate stationary threat signal sources in 3D space, using only azimuth and elevation measurements, and a single aerial platform. The proposed solutions are intended to be useful in applications such as ES missions, where the capability to locate ground-based threats and targets is crucial to survival and mission effectiveness. For an efficient performance at the tactical level of battlefield operations, the most important system requirements are rapid deployment, real- or near-real-time interception, and geolocation of threat signals, in addition to low size, weight, and power (SWaP) characteristics [27]. Radar warning receivers (RWRs), widely used by manned aircraft for electronic intelligence (ELINT), and DF seekers, which are more accurate sensor systems widely used by UASs [28], require a horizontal antenna array to generate azimuth measurements and a vertical array to obtain elevation measurements.

In this study, three batch-processing algorithms and one instantaneous solution were developed and investigated by simulations. The performance was degraded if only a few observation points were available. Increasing the observation points improved the position estimation accuracy. The proposed solutions can also serve as a launching point for further improvements and developments to meet evolving and changing requirements, e.g., integration into larger ES systems.

The main contribution of this article is the proposal and performance investigation of batch-processing and instantaneous passive emitter geolocation estimation algorithms, in 3D space, based on LS and particle filter (PF) estimation, with only azimuth and elevation measurements, and a single aerial platform, i.e., as few resources as possible, for ES applications.

The rest of the article is organized as follows: Section 2 reviews the main angle-based geolocation techniques used in ES missions, and Section 3 describes the main AoA estimation techniques. Three-dimensional positioning algorithms using azimuth and elevation measurements are developed in Section 4. Simulation results are presented in Section 5, and conclusions are given in Section 6.

## 2. Angle-Based Geolocation Techniques for Electronic Support Missions

The main threats to the survival of aircraft on modern battlefields are radar surveillance and radar-directed weapons. RWRs are designed to counter these threat sources by providing vital and timely information on the signal environment to the pilot and aircrew. The RWR system is an example of an ES system (complex RWRs are also referred to as ES systems) and illustrates the electronic order of battle (EOB) that immediately impacts the survival of a single aircraft. Therefore, the RWR is the heart of aircraft survivability equipment (ASE). RWRs usually deliver rough estimates of threat positions and ranges, while ES systems have more significant DF capabilities and, hence, provide more accurate emitter location estimates [11]. Threat geolocation systems, which are a step above RWR systems, can deliver accurate threat position information for many aircraft over an entire area to enable threat avoidance, suppression, or preemptive attacking of adversary radar sites [29]. RWRs provide accurate threat location information when the aircraft is flying straight and level, or is maneuvering up to certain limits of bank angle and turn rate. Exceeding these limits makes threat position information unreliable. The performance of RWRs is degraded or limited by the impact of electromagnetic interference (EMI), which depends on the signal environment. Main EMI impact sources include noise and deception jamming. Fighter aircraft RWR systems often use four antennas to provide 360° angular coverage, while naval RWR systems often use six or eight antennas.

Threat geolocation puts a defined position, i.e., coordinates, on a threat source to simply warn about the threat or to enable targeting and attacking of the threat, if the position information is accurate enough. Threat positioning can be performed earlier only by specialized tactical aircraft or strategic assets. Mobile threat sources move and relocate continuously. Therefore, position information of mobile threats delivered by strategic channels, with inherent time delays, is often obsolete. The basic geolocation, also referred to as DF, techniques include angle-based (i.e., triangulation), phase-based, (i.e., interferometry), and time-based, (i.e., ToA and TDoA) approaches. These techniques enable rapid positioning of radar threats, e.g., emitting radar on-board tactical assets. The performance of these techniques is heavily dependent on the accurate positioning of the receiving aircraft, which is made easy by using global navigation satellite system (GNSS) receivers.

Triangulation, also known as AoA or direction of arrival (DoA), involves taking direction, i.e., azimuth (horizontal angular), measurements from more than one position. The intersection of these measurements, also called lines of bearing (LoBs), is the 2D location estimate of the threat source, i.e., the enemy emitter. The best azimuth cuts are those that approach a right angle. Instantaneous (single pulse or monopulse) 3D positioning of threat sources requires an elevation (vertical angular) measurement, an estimate of the local equivalent (effective) height of the ionosphere where the signals are reflected, or a digital map of the local area to determine the range to the threat source, in addition to an azimuth measurement [30] (pp. 129–130). The 3D positioning technique using azimuth, elevation, and ionospheric information can be accomplished using a single sensor aboard a single platform. Therefore, it is also referred to as a single-site location (SSL). All AoA estimation methods are based on measuring the TDoA or phase difference of the signals from two antennas spaced half a wavelength or less apart [31]. To avoid ambiguities, at least two of the involved set of antennas have to meet this requirement.

Triangulation can be carried out by a set of aircraft equipped with RWR systems or by a single aircraft over time. The increased angle-off and the speed of interception are the main advantages of employing multiple aircraft. The major disadvantages of multi-aircraft triangulation are the need to determine optimal trajectories to improve the geometric dilution of precision (GDoP) conditions for accurate geolocation, the need to exchange measurements via a suitable low bandwidth communication network, and the need for an association procedure to ensure that all platforms are measuring the same signal, which is a difficult task in dense signal environments. Single-aircraft triangulation eliminates the measurement transfer requirement, but the aircraft needs to transmit some distance, on an optimal trajectory when possible, to get multiple azimuth measurements in which their cuts, preferably, approach a right angle. It is also a requirement that all measurements belong to the same threat source. Additional factors that impact the accuracy of DF operations include the quality of the equipment, the distance to the threat radar or source, the aircraft velocity, and the time length of the emitting threat signal.

Further location estimation methods involve phase rate of change measurements and hybrid angle-distance techniques. The phase rate of change is a triangulation method in which calculations are made using the phase derivative. The angle-distance approach can derive the rough distance or range to the threat signal source from RSS measurements and threat signal characteristics if the threat signal source is correctly identified and classified. The one-way link equation formula utilizes the equivalent radiated power (ERP) of the identified emitter to calculate the approximate range. Techniques based on azimuth angle and distance (or range) measurements are monopulse methods, i.e., can be accomplished using a single platform, and are commonly used by RWRs with limited RMS accuracy of about 15° of the angle and 25% of the range [30] (p. 132). The angle inaccuracy is mainly due to reflections from the platform that decrease the accuracy of antenna patterns and, hence, the resolution of amplitude comparison as described in Section 3. Ranging inaccuracies are mainly caused by propagation losses and the ERP of the identified emitter, which might be inaccurate [30] (p. 132).

## 3. Main Angle of Arrival Estimation Techniques

There are several methods for DF (i.e., AoA, DoA estimation, or LoB) of emitted signals. DF methods have different sets of features and restrictions. In increasing the order of both accuracy and complexity, AoA estimations can be obtained by utilizing the directional properties of single antennas, sophisticated configurations of closely spaced antennas, or phased arrays [9,32,33]. Brief reviews of DF systems using AoA measurements are given in [9] for ease of comparison, and in [34,35] with operational context. The main AoA measurement systems for triangulation are beam pattern-based DF, i.e., scanning beam, amplitude comparison, phase interferometry, and Doppler-based DF [9,11].

Searching for the bearing that maximizes the received signal power is the simplest DF method [9]. This beam pattern-based DF utilizes antennas with non-uniform responses to signals at different angles of arrival. Adcock antennas combine two omnidirectional antennas via subtraction to approximate a cosine pattern, providing a large field of view at poor resolution. They are often used as part of more complex DF systems [9]. The scanning beam requires only a single receiver, and it has high sensitivity due to employing a directive antenna. The slow response of the mechanically scanning beam is due to the rotation through the whole coverage angle to guarantee the interception of adversary emitters. Moreover, if the adversary emitter employs a scanning directional antenna, both beams must point at each other to maximize sensitivity, which may happen but at a low probability. This effect, in addition to the slow response, renders scanning beam techniques to a low probability of intercept (PoI) [11].

Amplitude comparison, also known as simultaneous multiple beams, is a very common, simple, reliable, low-cost, and small-sized system, which has relatively poor sensitivity and low resolution (accuracy) and a root mean square (RMS) of 3° to 10°. The Watson-Watt amplitude comparison technique consists of two different two-element Adcock antennas that are directed 90° from each other to compare two orthogonal cosine patterns [9]. Watson-Watt receivers achieve an AoA estimation accuracy of 2.5° with a sufficient signal-to-noise ratio (SNR) [35].

Phase interferometry, also known as PDoA, works by comparing the phase information of a threat signal wave between two or more DF antennas after collecting coherent complex measurements at them [9,36,37]. The resulting phase difference is then used to generate an AoA measurement. DF antennas are installed at different locations on an aircraft and multiple AoA measurements are used to estimate the position of the threat signal. Employing multiple antennas in an array setup allows for carrying out simultaneous azimuth and elevation measurements to rapidly localize the threat emitter in 3D space. A phased interferometer or an array is a high-cost and large-size system with a very high-resolution and an RMS of 0.1° to 3° [11]. Most RWR systems use a four-quadrant amplitude comparison for DF, where only one antenna per quadrant is usually employed to cover the 2 to 18 GHz band. Sensitivity and accuracy can be improved by increasing the number of antennas, e.g., employing eight antennas would provide 3 dB more gain and double the accuracy [11].

Doppler-based DF compares received frequencies at a stationary reference antenna and a test antenna while rotating around the reference to obtain AoA estimations. Stationary test antennas, arranged in a ring around the reference antenna, can be alternatively employed to mimic a rotating antenna by rapidly switching them. The AoA estimation performance of Doppler DF systems is similar to that of the Watson-Watt techniques [9].

The performance of DF methods is usually assessed by the standard deviation (SD) of AoA estimation and the angular resolution [9], i.e., the minimum angular separation between any two signal sources that can assist in resolving them. Multipath signal propagation leads to incorrect AoA measurements or ghost targets. AoA measurement deficiencies due to, for example, incorrect measurement association of multiple adversary sources, are addressed by providing additional and side-channel information about parameters of the received signal. However, measurement association in the presence of a multipath signal is an extremely challenging problem [9]. AoA estimation relies on the adversary source emitting signals, i.e., it is vulnerable to the source’s radio silence. If enemy sources are not emitting signals or are emitting in a frequency band that is not covered by the AoA system, they will neither be detected nor localized. If the adversary can inject false data, or corrupt signal information [38], the performance of AoA-based geolocation is devastated. Studying this problem and providing methods to mitigate its impact is an open research area [9].

## 4. Positioning Algorithms Using Azimuth and Elevation Measurements

The mathematical formulation for estimating an emitter location, i.e., the positioning problem, in 3D space using azimuth and elevation measurements will be presented first. Then, three batch-processing solution algorithms, Section 4.1, Section 4.2 and Section 4.3, and an instantaneous geolocation solution, Section 4.4, will be developed and discussed.

**Figure 1:**
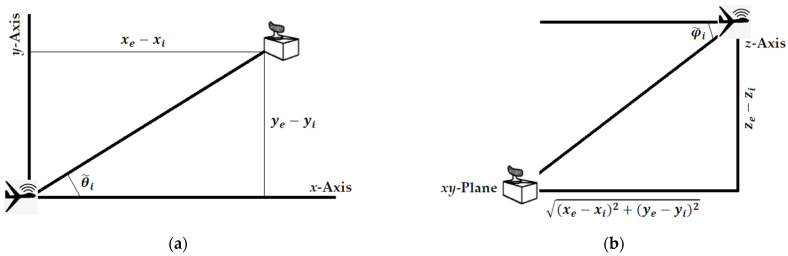
Geometrical illustration of the obtained angle measurements: (**a**) azimuth; and (**b**) elevation.

For the following, let $p_{e}={\left[\begin{array}{ccc} x_{e} & y_{e} & z_{e} \end{array}\right]}^{T}$ be the unknown 3D position of the emitter, and $r_{i}={\left[\begin{array}{ccc} x_{i} & y_{i} & z_{i} \end{array}\right]}^{T}$ be the known 3D position of the ES sensor or receiver at the measurement (i.e., observation) point i=1, 2, …, N in a 3D Cartesian coordinate system, while $\theta_{i}$ and $\varphi_{i}$ are, respectively, the error-free azimuth and elevation measurements. The azimuth and elevation measurements are obtained by the horizontal and vertical antenna arrays, respectively, of the ES sensor system aboard the aerial platform (see Figure 1). Assuming zero-mean Gaussian noise, the azimuth and elevation noisy measurements can be modeled, respectively, as [9] (pp. 200, 202):

(1) $${\tilde{\theta }}_{i}=\tan^{-1}\left(\frac{y_{e}-y_{i}}{x_{e}-x_{i}}\right)+n_{\theta },-\pi <{\tilde{\theta }}_{i}\leq \pi ,\;i=1,\;2,\;\ldots ,\;N,$$

and

(2) $${\tilde{\varphi }}_{i}=\tan^{-1}\left(\frac{z_{e}-z_{i}}{\sqrt{{\left(x_{e}-x_{i}\right)}^{2}+{\left(y_{e}-y_{i}\right)}^{2}}}\right)+n_{\varphi },-\frac{\pi }{2}<{\tilde{\varphi }}_{i}<\frac{\pi }{2},\;i=1,\;2,\;\ldots ,\;N,$$

where $n_{\theta }$, $n_{\theta }\sim N\left(0,\sigma_{\theta }^{2}\right)$, and $n_{\varphi }$, $n_{\varphi }\sim N\left(0,\sigma_{\varphi }^{2}\right)$, are the azimuth and elevation measurement noise or errors, with variances $\sigma_{\theta }^{2}$ and $\sigma_{\varphi }^{2}$, respectively. The assumed error models are sufficient for geolocation when the ES sensor system is properly calibrated. Equation (1) is solved by the four-quadrant inverse tangent, e.g., the atan2 function available in MATLAB, while Equation (2) is solved by the inverse tangent, e.g., the MATLAB atan function. Equations (1) and (2) represent simplified sensor models appropriate to demonstrate and investigate the performance of the algorithms proposed in this study. The models skip all signal processing details and output noisy azimuth and elevation measurements. The problem, thus, is to estimate the vector $p_{e}$ given a set of noisy measurements, ${\tilde{\theta }}_{i}$ and ${\tilde{\varphi }}_{i}$, and using the known vectors $r_{i}$, which might, in turn, contain uncertainties.

### 4.1. Two-Step Closed-Form Solution

By multiplying out terms in Equation (1) and rearranging while neglecting noise we get [39]:

(3) $$x_{e}\sin{\tilde{\theta }}_{i}-y_{e}\cos{\tilde{\theta }}_{i}\approx x_{i}\sin{\tilde{\theta }}_{i}-y_{i}\cos{\tilde{\theta }}_{i},\;i=1,\;2,\;\ldots ,\;N.$$

Stacking all the information in Equation (3), we obtain [39]:

(4) $$\left[\begin{array}{cc} \sin{\tilde{\theta }}_{1} & -\cos{\tilde{\theta }}_{1}\\ \vdots & \vdots \\ \sin{\tilde{\theta }}_{N} & -\cos{\tilde{\theta }}_{N} \end{array}\right]\left[\begin{array}{c} x_{e}\\ y_{e} \end{array}\right]\approx \left[\begin{array}{c} x_{1}\sin{\tilde{\theta }}_{1}-y_{1}\cos{\tilde{\theta }}_{1}\\ \vdots \\ x_{N}\sin{\tilde{\theta }}_{N}-y_{N}\cos{\tilde{\theta }}_{N} \end{array}\right].$$

The matrix-vector notation for Equation (4) is written as [39]:

(5) $$H\left(\theta \right)p_{e}^{2D}\approx b\left(\theta \right).$$

The LS estimate of the horizontal emitter location, ${\hat{p}}_{e}^{2D}$, which can also be found in [19], is defined by [39]:

(6) $${\hat{p}}_{e}^{2D}={\left(H{\left(\theta \right)}^{T}H\left(\theta \right)\right)}^{-1}H\left(\theta \right)b\left(\theta \right).$$

Multiplying out terms in Equation (2) and rearranging while neglecting noise we get:

(7) $$z_{e}\approx z_{i}+\sqrt{{\left(x_{e}-x_{i}\right)}^{2}+{\left(y_{e}-y_{i}\right)}^{2}}\tan{\tilde{\varphi }}_{i},\;i=1,\;2,\;\ldots ,\;N.$$

An estimate of the vertical emitter location, ${\hat{z}}_{e}$, is calculated as the mean value of all information contained in Equation (7) by:

(8) $${\hat{z}}_{e}=\frac{1}{N}\sum_{i=1}^{N}\left(z_{i}+\sqrt{{\left(x_{e}-x_{i}\right)}^{2}+{\left(y_{e}-y_{i}\right)}^{2}}\tan{\tilde{\varphi }}_{i}\right).$$

The 3D estimate of the emitter location, ${\hat{p}}_{e}$, is obtained in two steps: (1) estimating ${\hat{p}}_{e}^{2D}$ by a 2D CF LS algorithm using ${\tilde{\theta }}_{i}$, $x_{i}$, and $y_{i}$ as defined by Equation (6); and (2) using ${\hat{p}}_{e}^{2D}$, $r_{i}$, and ${\tilde{\varphi }}_{i}$ as indicated by Equation (8) to estimate ${\hat{z}}_{e}$.

### 4.2. Single-Step Closed-Form Solution

Let $s_{i}^{2D}=\sqrt{{\left(x_{e}-x_{i}\right)}^{2}+{\left(y_{e}-y_{i}\right)}^{2}}$. The terms in Equation (2) can be multiplied out and rearranged while neglecting noise to obtain:

(9) $$s_{i}^{2D}\sin{\tilde{\varphi }}_{i}\approx \left(z_{e}-z_{i}\right)\cos{\tilde{\varphi }}_{i},\;i=1,\;2,\;\ldots ,\;N.$$

Since $s_{i}^{2D}\approx \left(x_{e}-x_{i}\right)\cos{\tilde{\theta }}_{i}+\left(y_{e}-y_{i}\right)\sin{\tilde{\theta }}_{i}$, Equation (9) can be rewritten as:

(10) $$x_{e}\sin{\tilde{\varphi }}_{i}\cos{\tilde{\theta }}_{i}+y_{e}\sin{\tilde{\varphi }}_{i}\sin{\tilde{\theta }}_{i}-z_{e}\cos{\tilde{\varphi }}_{i}\approx x_{i}\sin{\tilde{\varphi }}_{i}\cos{\tilde{\theta }}_{i}+y_{i}\sin{\tilde{\varphi }}_{i}\sin{\tilde{\theta }}_{i}-z_{i}\cos{\tilde{\varphi }}_{i},\;i=1,\;2,\;\ldots ,\;N.$$

Stacking all the information in Equations (3) and (10), we get:

(11) $$\left[\begin{array}{ccc} \sin{\tilde{\theta }}_{1} & -\cos{\tilde{\theta }}_{1} & 0\\ \sin{\tilde{\varphi }}_{1}\cos{\tilde{\theta }}_{1} & \sin{\tilde{\varphi }}_{1}\sin{\tilde{\theta }}_{1} & -\cos{\tilde{\varphi }}_{1}\\ \vdots & \vdots & \vdots \\ \sin{\tilde{\theta }}_{N} & -\cos{\tilde{\theta }}_{N} & 0\\ \sin{\tilde{\varphi }}_{N}\cos{\tilde{\theta }}_{N} & \sin{\tilde{\varphi }}_{N}\sin{\tilde{\theta }}_{N} & -\cos{\tilde{\varphi }}_{N} \end{array}\right]\left[\begin{array}{c} x_{e}\\ y_{e}\\ z_{e} \end{array}\right]\approx \left[\begin{array}{c} x_{1}\sin{\tilde{\theta }}_{1}-y_{1}\cos{\tilde{\theta }}_{1}\\ x_{1}\sin{\tilde{\varphi }}_{1}\cos{\tilde{\theta }}_{1}+y_{1}\sin{\tilde{\varphi }}_{1}\sin{\tilde{\theta }}_{1}-z_{1}\cos{\tilde{\varphi }}_{1}\\ \vdots \\ x_{N}\sin{\tilde{\theta }}_{N}-y_{N}\cos{\tilde{\theta }}_{N}\\ x_{N}\sin{\tilde{\varphi }}_{N}\cos{\tilde{\theta }}_{N}+y_{N}\sin{\tilde{\varphi }}_{N}\sin{\tilde{\theta }}_{N}-z_{N}\cos{\tilde{\varphi }}_{N} \end{array}\right].$$

The matrix-vector notation for Equation (11) is written as:

(12) $$H\left(\theta ,\varphi \right)p_{e}\approx b\left(\theta ,\varphi \right).$$

The LS estimate of the emitter location, ${\hat{p}}_{e}$, is defined by:

(13) $${\hat{p}}_{e}={\left(H{\left(\theta ,\varphi \right)}^{T}H\left(\theta ,\varphi \right)\right)}^{-1}H\left(\theta ,\varphi \right)b\left(\theta ,\varphi \right).$$

### 4.3. Particle Filter Solution

The PF [40] considers the emitter location, i.e., the state, $p_{e}$, as a random variable and estimates it by using random samples called particles, instead of parametric distributions, to overcome the limitations caused by the Gaussian assumption. Therefore, the PF can simultaneously deal with nonlinear models and non-Gaussian or multimodal distributions. The PF approximates the posterior probability density function (PDF), $p(p_{e}|Z)$, as a weighted combination of particles [41]:

(14) $$p(p_{e}|Z)\approx \sum_{p=1}^{P}w_{p}\delta \left(p_{e}-s_{p}\right),$$

where $Z={\left[\begin{array}{cc} {\tilde{\theta }}_{i} & {\tilde{\varphi }}_{i} \end{array}\right]}^{T}$, i=1, 2, …, N refers to all azimuth and elevation measurements, δ(x) is the Dirac delta function, $w_{p}$ is the weight of the particle, $s_{p}$, and P is the total number of particles. All weights sum up to unity to represent a valid posterior PDF.

The PF implementation is listed in Algorithm 1. To avoid evaluating an infinite continuous space and to concentrate the particles in the relevant space, the search space has to be limited as much as possible, while accounting for measurement uncertainties and including the true emitter location. This is achieved by doing the following calculations at each measurement point i=1, 2, …, N.

Assuming $z_{e}=0$ in Equation (2), a rough horizontal range estimate, ${\hat{s}}_{i}^{2D}\approx \sqrt{{\left(x_{e}-x_{i}\right)}^{2}+{\left(y_{e}-y_{i}\right)}^{2}}$, at the measurement point i can be obtained as:

(15) $${\hat{s}}_{i}^{2D}\approx -\frac{z_{i}}{\tan{\tilde{\varphi }}_{i}}.$$

The components of ${\hat{s}}_{i}^{2D}$ in the *x*- and *y*-directions, $lx_{i}$ and $ly_{i}$, are, respectively, defined by:

(16) $$lx_{i}=\left|{\hat{s}}_{i}^{2D}\right|\cos{\tilde{\theta }}_{i},$$

and

(17) $$ly_{i}=\left|{\hat{s}}_{i}^{2D}\right|\sin{\tilde{\theta }}_{i}.$$

To account for uncertainties, i.e., measurement errors and the assumption $z_{e}=0$, ranging uncertainties in the *x*- and *y*-directions, $rx_{i}$ and $ry_{i}$, are, respectively, determined as:

(18) $$rx_{i}=\alpha \cdot lx_{i},$$

and

(19) $$ry_{i}=\alpha \cdot ly_{i},$$

where α is a calibration parameter, set to 0.25 in the PF implementation, to ensure that the horizontal search area of the PF will include the true horizontal emitter location.

The search space considered for the PF was a cuboid domain, which is suitable for 3D Cartesian coordinate systems, and must contain the true emitter position. For convenience, it was assumed that $\cos{\tilde{\theta }}_{i}$ and $\sin{\tilde{\theta }}_{i}$ were positive values, and that the receiver was above the emitter. For arbitrary geometries, the computations below have to be adjusted appropriately, e.g., if $\cos{\tilde{\theta }}_{i}$ is a negative value, the plus sign of the second term in Equations (20) and (21) is changed to a minus sign. The cuboid is limited in the *x*-direction at each measurement point i, in the range between $Vx_{i}^{min}$ and $Vx_{i}^{max}$, which are defined, respectively, by:

(20) $$Vx_{i}^{min}=x_{i}+lx_{i}-rx_{i}.$$

and

(21) $$Vx_{i}^{max}=x_{i}+lx_{i}+rx_{i}.$$

In the *y*-direction, the cuboid is limited in the range between $Vy_{i}^{min}$ and $Vy_{i}^{max}$, defined, respectively, by:

(22) $$Vy_{i}^{min}=y_{i}+ly_{i}-ry_{i},$$

and

(23) $$Vy_{i}^{max}=y_{i}+ly_{i}+ry_{i}.$$

The cuboid is limited in the *z*-direction in the range between $Vz_{i}^{min}$ and $Vz_{i}^{max}$, defined, respectively, by:

(24) $$Vz_{i}^{min}=0,$$

and

(25) $$Vz_{i}^{max}=z_{i},$$

since the UAS collecting the measurements is above the ground threat emitter.

When all azimuth and elevation measurements are collected, the mean values of Equations (20)–(25), representing the mean dimensions of the search cuboid, denoted as $Vx^{min}$, $Vx^{max}$, $Vy^{min}$, $Vy^{max}$, $Vz^{min}$, and $Vz^{max}$, respectively, are computed. Thus, a set of P particles, $s_{p}={\left[\begin{array}{ccc} x_{p} & y_{p} & z_{p} \end{array}\right]}^{T}$, p=1, 2, …, P, are generated by a uniform distribution within the cuboid as:

(26) $$x_{p}\sim U\left(Vx^{min},\;Vx^{max}\right),$$

(27) $$y_{p}\sim U\left(Vy^{min},\;Vy^{max}\right),$$

and

(28) $$z_{p}\sim U\left(Vz^{min},\;Vz^{max}\right).$$

Equations (26)–(28), thus, represent the prior distribution of the random variable $p_{e}$. For each particle, $s_{p}$, azimuth and elevation predictions, i.e., $\theta_{i}^{p}$ and $\varphi_{i}^{p}$, at any measurement point are computed, respectively, as:

(29) $$\theta_{i}^{p}=\tan^{-1}\frac{y_{p}-y_{i}}{x_{p}-x_{i}},$$

and

(30) $$\varphi_{i}^{p}=\tan^{-1}\frac{z_{p}-z_{i}}{\sqrt{{\left(x_{p}-x_{i}\right)}^{2}+{\left(y_{p}-y_{i}\right)}^{2}}}.$$

The weight, $w_{p}$, of each particle, $s_{p}$, is then computed as:

(31) $$w_{p}=\eta \cdot {\left(\sum_{i=1}^{N}{\left({\tilde{\theta }}_{i}-\theta_{i}^{p}\right)}^{2}+\sum_{i=1}^{N}{\left({\tilde{\varphi }}_{i}-\varphi_{i}^{p}\right)}^{2}\right)}^{-1}.$$

where $\eta ={\left(\sum_{p=1}^{P}w_{p}\right)}^{-1}$ is a normalizing factor to ensure a valid posterior PDF, i.e., all weights sum up to unity.

The weight of each particle is inversely proportional to the similarity metric used, which is the sum of squared distances between the measured and predicted azimuth and elevation angles. The implementation of Equation (31) has to account for the rare case of getting zero values for the two summation terms to avoid division by zero. The estimate of the emitter location, ${\hat{p}}_{e}$, is obtained by the weighted trimmed average estimate (WTAE) [41,42], in which a number L, L<P, of the best-weighted particles, is selected and their weights are normalized as:

(32) $${\hat{p}}_{e}=\frac{\sum_{p=1}^{L}s_{p}w_{p}}{\sum_{p=1}^{L}w_{p}}.$$

where 2% of the best-weighted particles are used in implementing Equation (32), i.e., L=0.02·P.
**Algorithm 1.** PF algorithm for 3D emitter location using azimuth and elevation measurements.1. At each measurement point i=1, 2, …, N, calculate the parameters necessary to determine the mean dimensions of the cuboid search space, i.e., $Vx^{min}$, $Vx^{max}$, $Vy^{min}$, $Vy^{max}$, $Vz^{min}$, and $Vz^{max}$, by solving Equations (15)–(25);2. After collecting all azimuth and elevation angle measurements, generate a set of P particles uniformly distributed within the cuboid search space, according to Equations (26)–(28);3. Compute azimuth and elevation angle predictions, i.e., $\theta_{i}^{p}$ and $\varphi_{i}^{p}$, for each particle, $s_{p}$, according to Equations (29) and (30);4. Compute the weight, $w_{p}$, of each particle, $s_{p}$, according to Equation (31);5. Obtain the estimate of the emitter location, ${\hat{p}}_{e}$, by Equation (32).

**Figure 2:**
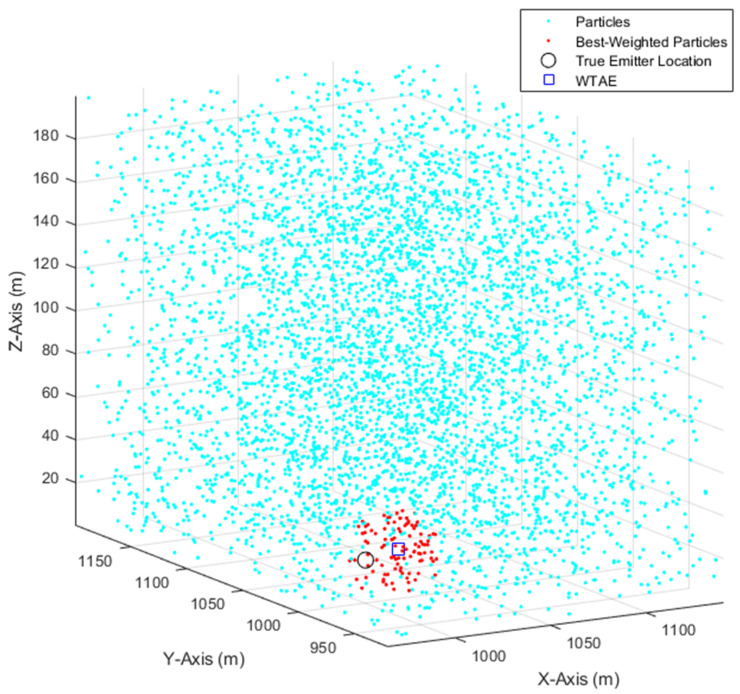
Illustration of the PF implementation.

The implementation of Equations (26)–(28), with 5000 particles, when a UAS moved along a linear path collecting 101 measurements, with $\sigma_{\theta }=\sigma_{\varphi }=3{}^{\circ}$ is illustrated in Figure 2, along with the best-weighted particles and WTAE of the emitter location in Equation (32).

### 4.4. Instantaneous Particle Filter Solution

Monopulse, i.e., instantaneous, solutions are necessary for certain applications, in which long-time observation of the threat source is infeasible or inadvisable. In the monopulse technique, a single pulse emitted by a threat source is sufficient for geolocation or DF. The accuracy can be improved by using several platforms simultaneously or by taking several measurements successively over time at different locations using a single platform. Therefore, the performance of monopulse techniques is severely impaired in bad signal conditions, e.g., multipath propagation.

The instantaneous particle filter (IPF), listed in Algorithm 2, performs the calculations of Equations (15)–(25) to determine the cuboid search space necessary to estimate the emitter location if a single pulse is emitted by the threat source, i.e., i=1, and the first azimuth and elevation measurements, $\theta_{1}$ and $\varphi_{1}$, are available. A set of P particles, $s_{p}={\left[\begin{array}{ccc} x_{p} & y_{p} & z_{p} \end{array}\right]}^{T}$, p=1, 2, …, P, are then generated by a uniform distribution within the cuboid as:

(33) $$x_{p}\sim U\left(Vx_{i}^{min},\;Vx_{i}^{max}\right),$$

(34) $$y_{p}\sim U\left(Vy_{i}^{min},\;Vy_{i}^{max}\right),$$

and

(35) $$z_{p}\sim U\left(Vz_{i}^{min},\;Vz_{i}^{max}\right).$$

The IPF continues the procedures according to Equations (29)–(32), where i=1, to obtain the first emitter location estimate. The IPF considers 10% of the best-weighted particles, i.e., L=0.1·P, in Equation (32). If additional measurements can be obtained by the UAS, i.e., i>1, the first emitter location estimate can be improved by adjusting the cuboid domain before evaluating the new measurements.
**Algorithm 2.** IPF algorithm for 3D emitter location using azimuth and elevation measurements.1. If at least a single pulse is emitted by the threat source and the first azimuth and elevation measurements, $\theta_{1}$ and $\varphi_{1}$, are available, solve Equations (15)–(25) to determine the cuboid search space, where i=1;2. Generate a set of P particles uniformly distributed within the cuboid search space, according to Equations (33)–(35);3. Compute azimuth and elevation angle predictions, i.e., $\theta_{1}^{p}$ and $\varphi_{1}^{p}$, for each particle, $s_{p}$, according to Equations (29) and (30), where i=1;4. Compute the weight, $w_{p}$, of each particle, $s_{p}$, according to Equation (31);5. Obtain the first estimate of the emitter location, ${\hat{p}}_{e}$, by Equation (32);6. If more measurements are available, i.e., i>1;
7. Test whether the recent emitter location estimate, ${\hat{p}}_{e,i-1}$, is within the new cuboid search space determined by Equations (15)–(25);8. If the test is positive, move the center of the search cuboid to ${\hat{p}}_{e,i-1}$, and generate a set of P particles uniformly distributed within the cuboid search space, according to Equations (36)–(38);9. If the test failed in any of the directions, the particles are generated within the cuboid search space in the respective direction according to the corresponding distribution, from Equations (33)–(35);10. Execute steps 3–5, where i>1.

Firstly, the IPF tests whether the recent emitter location estimate, ${\hat{p}}_{e,i-1}$, i>1, is within the new cuboid search space, via Equations (15)–(25). If the test is positive in the *x*-, *y*-, and *z*-directions, the center of the search cuboid is moved to the recent emitter location estimate, and the domain of the search cuboid is shrunk to put more focus on the potential search space. Thus, a set of P particles, $s_{p}={\left[\begin{array}{ccc} x_{p} & y_{p} & z_{p} \end{array}\right]}^{T}$, p=1, 2, …, P, are generated by a uniform distribution within the new cuboid as:

(36) $$x_{p}\sim U\left(x_{e,i-1}-R_{x},\;x_{e,i-1}+R_{x}\right),\;i>1,$$

(37) $$y_{p}\sim U\left(y_{e,i-1}-R_{y},\;y_{e,i-1}+R_{y}\right),\;i>1,$$

and

(38) $$z_{p}\sim U\left(z_{e,i-1}-R_{z},\;z_{e,i-1}+R_{z}\right),\;i>1,$$

where $R_{x}$, $R_{y}$, and $R_{z}$, are the cuboid’s dimension limiting factors in the *x*-, *y*-, and *z*-directions, and are set to 10, 10, and 5 m in the IPF implementation, respectively. Thus, the new search space is within a cuboid of dimensions 20×20×10 m.

**Figure 3:**
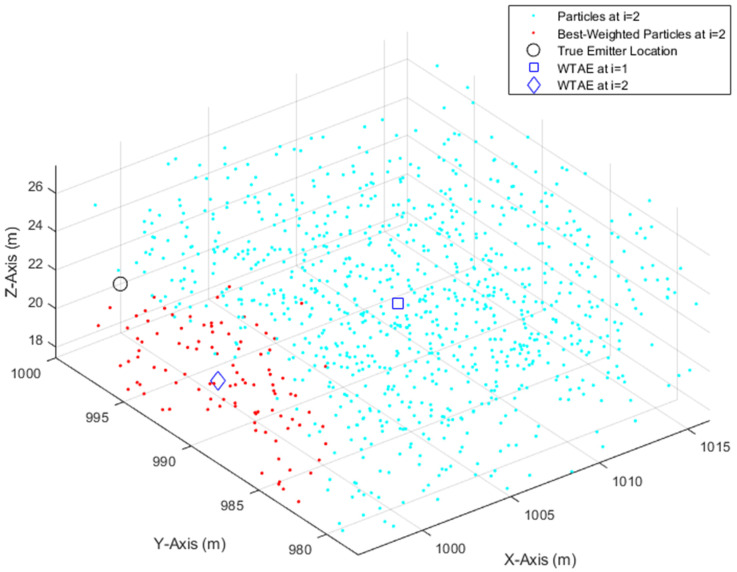
Illustration of the IPF implementation.

If the test failed in any of the three directions, the particles are generated within the search cuboid in the respective direction according to the corresponding distribution, from Equations (33)–(35), i>1, i.e., the cuboid dimension will not be shrunk in the direction where the test failed. Figure 3 illustrates the working of the IPF, with 1000 particles, after taking two measurements, with $\sigma_{\theta }=\sigma_{\varphi }=3{}^{\circ}$, by the UAS. The first emitter location estimate (blue square), computed by Equation (32), was computed by searching a cuboid domain with the dimensions as defined in Equations (33)–(35), and became the center of the second search cuboid with the shrunk dimensions as defined in Equations (36)–(38). It can be seen that the second emitter location estimate (blue diamond) is closer than the first estimate to the true emitter location.

## 5. Simulation Results

### 5.1. Assumptions and Simulation Setup

It was assumed that the aerial platform, i.e., UAS, was equipped with an ES sensor or DF seeker system to obtain azimuth and elevation measurements at different observation points, and that the target or emitter was detected, i.e., sufficient receiver sensitivity and sensor coverage to obtain the needed measurements were available. The single stationary emitter location case was considered. Thus, the data or measurement association problem, i.e., deghosting, accompanying the several-emitter location case was not present. Two different levels of capabilities can be provided: (1) basic DF information from very long ranges; and (2) geolocation capability at closer ranges. When a manned or unmanned aircraft starts from several kilometers away at a high altitude, it can only obtain general DF information, which helps with flying towards the threat emitter while also reducing the altitude, or commanding a small-sized, low-cost, less detectable, and high-survivable UAS to approach the threat at a lower altitude. Geolocation capability is achieved at close enough ranges. A simulation scenario with different measurement points and measurement accuracies was considered to demonstrate the geolocation capability.

**Figure 4:**
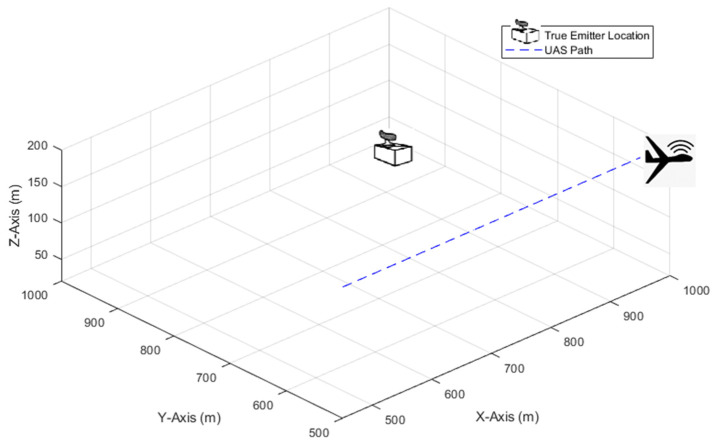
True emitter location and linear flight path of the UAS.

Most previous studies assume a LoB measurement accuracy of 1° to 2° [43] (p. 45). The performance of systems with higher inaccuracies is less well investigated. Typical DF accuracies of amplitude comparison and phase interferometer techniques are 3° to 10° and 0.1° to 3°, respectively [11]. In [27], a single system that can provide azimuth and elevation measurements with 2.5° accuracy was reported. Figure 4 illustrates the true emitter location and linear path of the UAS considered in the simulations. The emitter was located at (1000, 1000, 20) m and the UAS flew from (450, 500, 200) m to (950, 500, 200) m. Three different measurement points of 101, 51, and 11 were considered. In each case, the SDs of the azimuth and elevation measurement errors were assumed equal, i.e., $\sigma_{\theta }=\sigma_{\varphi }=\sigma_{\theta ,\varphi }$, and were varied from 1° to 5° in 1° steps. Low-pass filtering was applied to the measurements to reduce noise and remove outliers. Root mean square error (RMSE) results were obtained over 100 simulation runs for the considered measurement noise levels to evaluate the batch-processing solutions TSCF, SSCF, and PF, with 5000 particles (Section 5.2). Representative performances, in terms of the root square error (RSE), of the IPF (with 1000 particles) at the investigated measurement noise levels are illustrated in Section 5.3.

### 5.2. Results of the Batch-Processing Solutions

**Figure 5:**
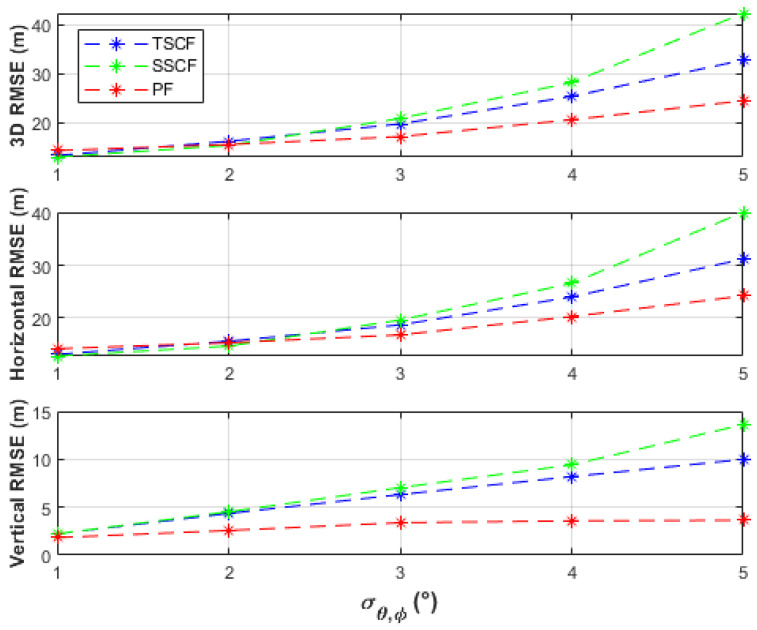
Performance comparison of the batch-processing solutions with certain UAS positions and using 101 measurement points.

The uncertainty of the UAS position, $\sigma_{p}=\sqrt{\sigma_{x}^{2}+\sigma_{y}^{2}+\sigma_{z}^{2}}$, were considered in the simulations. It was assumed that the uncertainties in the *x*-, *y*-, and *z*-directions were equal, i.e., $\sigma_{x}=\sigma_{y}=\sigma_{z}$. Uncertainty levels of $\sigma_{p}=0,\;1,\;5,\;\mathrm{and}\;10\;m$ were investigated. Figure 5 shows the 3D, horizontal, and vertical RMSE of the TSCF, SSCF, and PF solutions using azimuth and elevation measurements at 101 observation points, where $\sigma_{p}=0\;m$, i.e., certain knowledge about the UAS position was available. The results are also listed in Table 1.

**Figure 6:**
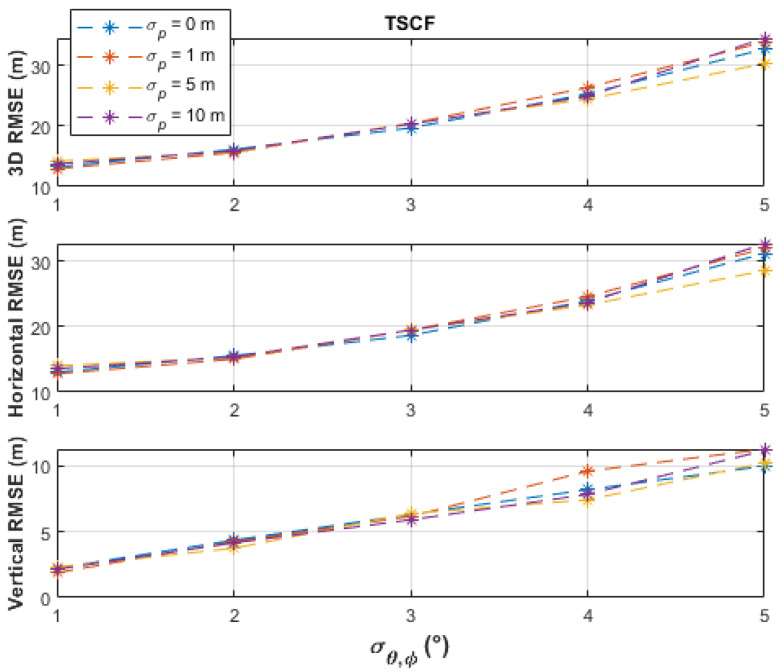
Performance comparison of the TSCF batch-processing solution under different levels of UAS position uncertainties and using 101 measurement points.

**Figure 7:**
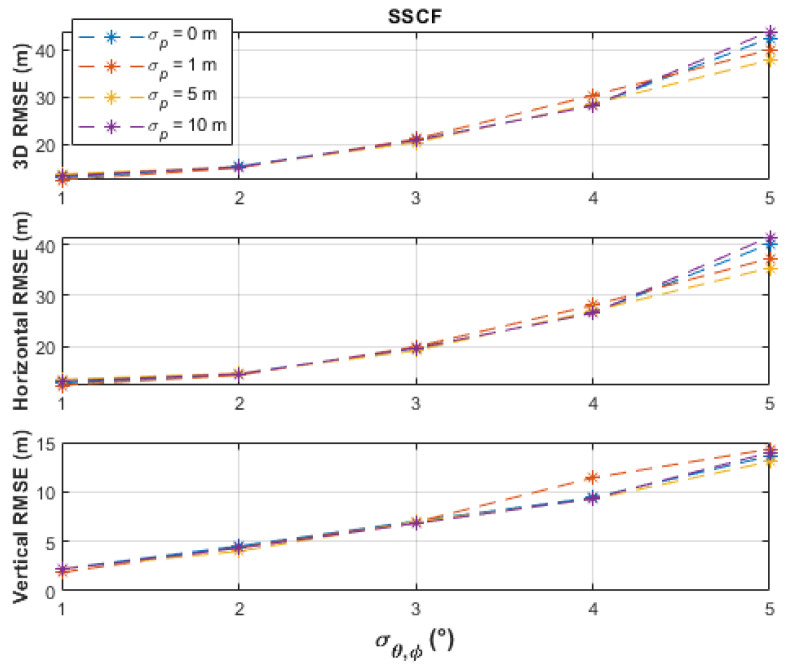
Performance comparison of the SSCF batch-processing solution under different levels of UAS position uncertainties and using 101 measurement points.

**Figure 8:**
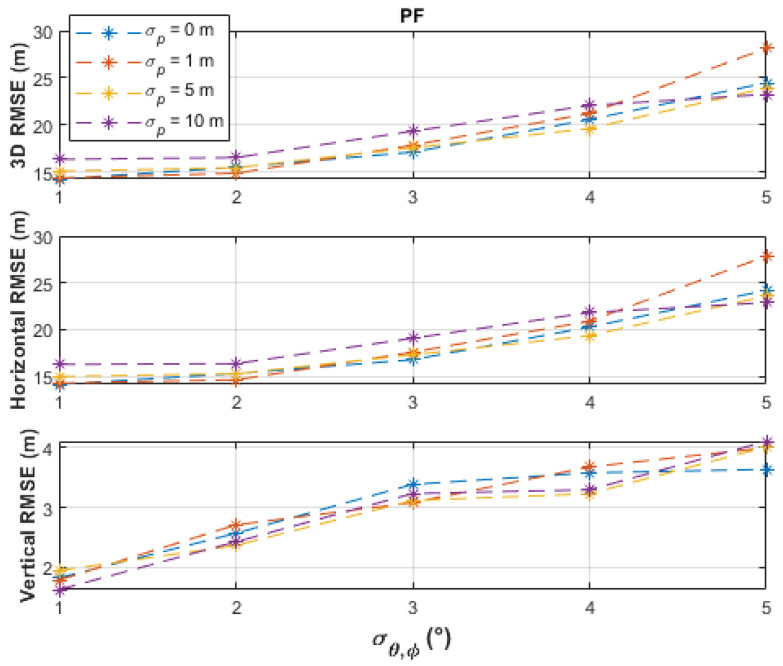
Performance comparison of the PF batch-processing solution under different levels of UAS position uncertainties and using 101 measurement points.

The batch-processing solutions have similar performances up to the measurement noise level, $\sigma_{\theta ,\varphi }$, of 2°, where less than 16 m horizontal RMSE and less than 5 m vertical RMSE were obtained. From a measurement noise level of 3°, the PF obtained the best horizontal accuracies (up to 24.19 m RMSE), followed by the TSCF solution (up to 31.22 m RMSE), and then by the SSCF solution (up to 40.12 m RMSE). The vertical RMSE of the PF was always under 4 m, better than the other two solutions, and was only slightly affected by varying the measurement noise level. The TSCF solution first estimated the horizontal emitter location, depending only on azimuth measurements, and then used this estimate together with the elevation measurements to estimate the vertical emitter location. Thus, the noisy azimuth and elevation measurements were dealt with separately. Therefore, the TSCF solution, at higher noise levels, was more accurate than the SSCF solution, in which terms with combined azimuth and elevation noisy measurements are additionally involved in the estimation process in Equation (11). Figure 6, Figure 7 and Figure 8 depict that the three algorithms, especially TSCF and SSCF, are quite robust against UAS position uncertainties.

**Figure 9:**
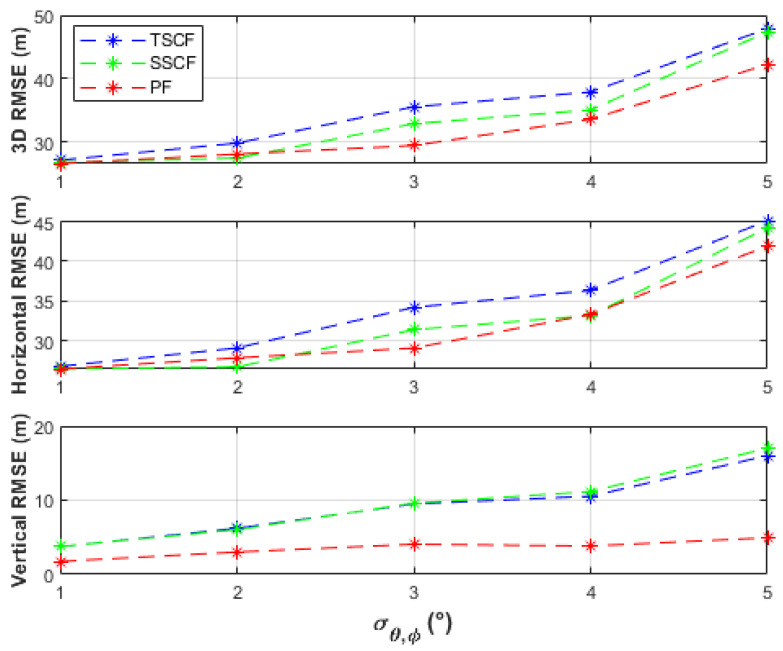
Performance comparison of the batch-processing solutions with certain UAS positions and using 51 measurement points.

The RMSE results, when using 51 measurement points, are shown in Figure 9 and listed in Table 2. Similar performance trends to the previous case can be observed, where higher RMSEs were obtained because fewer measurements were used. The PF still obtained the best performance, especially in the *z*-direction. The SSCF solution was slightly better than the TSCF solution. The advantage of processing the noisy azimuth and elevation measurements separately could not be fully exploited by the TSCF solution with fewer measurements. In general, the performance differences tended to be less significant. All solutions were also robust against UAS position uncertainties, and therefore the plots were skipped for brevity.

The RMSE results, with 11 measurement points and certain knowledge about the UAS position, are listed in Table 3. Emitter location accuracy was severely degraded if only a few measurements were available. Only the vertical emitter location estimates, obtained by the PF, were useful. The performance with UAS position uncertainties was similar, and therefore the results were also skipped. Actions to be taken by the UAS to improve performance include obtaining more measurements, obtaining measurements at closer ranges, or cooperating with other UASs and platforms.

### 5.3. Results of the Instantaneous Particle Filter Solution

**Figure 10:**
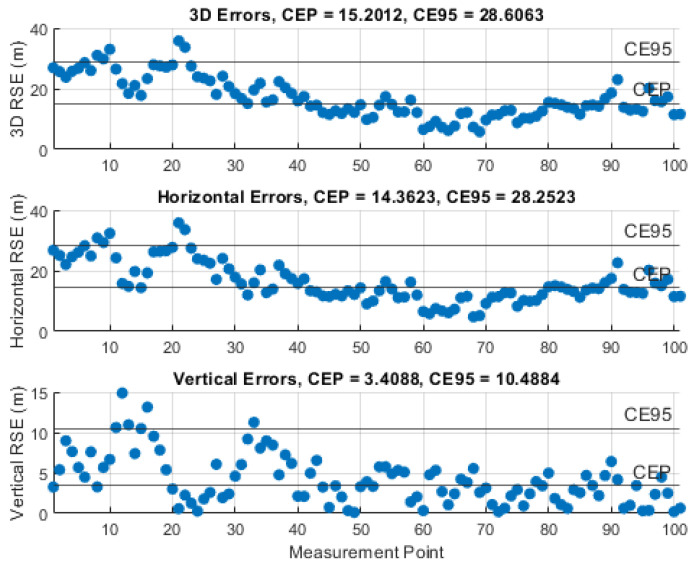
RSE evolution of the IPF solution with a measurement error SD, $\sigma_{\theta ,\varphi }$, of 1°.

**Figure 11:**
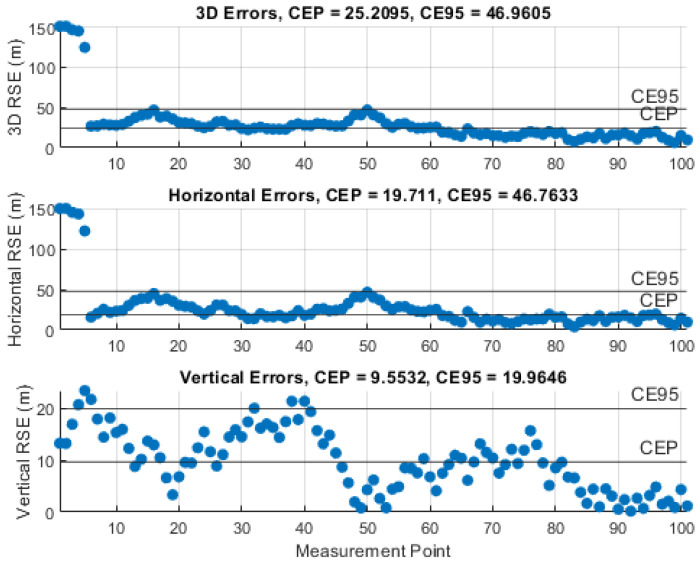
RSE evolution of the IPF solution with a measurement error SD, $\sigma_{\theta ,\varphi }$, of 2°.

**Figure 12:**
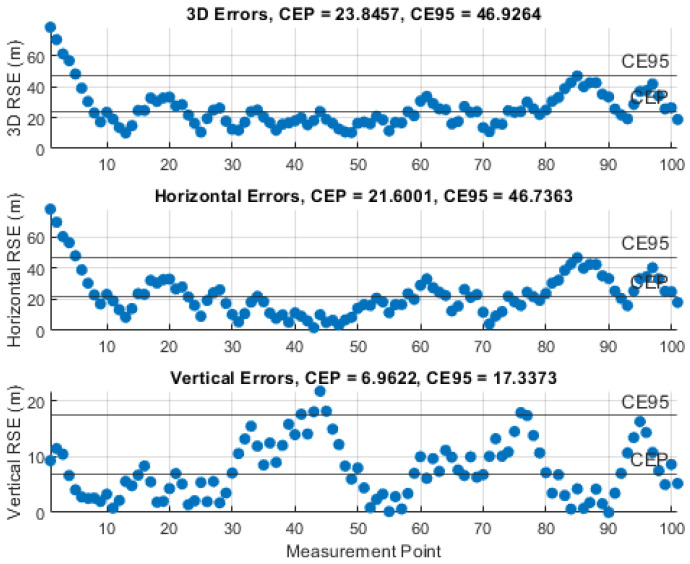
RSE evolution of the IPF solution with a measurement error SD, $\sigma_{\theta ,\varphi }$, of 3°.

**Figure 13:**
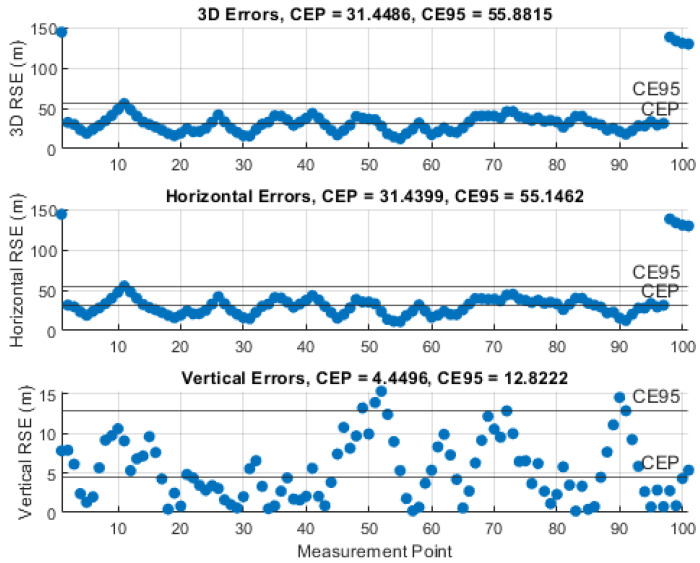
RSE evolution of the IPF solution with a measurement error SD, $\sigma_{\theta ,\varphi }$, of 4°.

**Figure 14:**
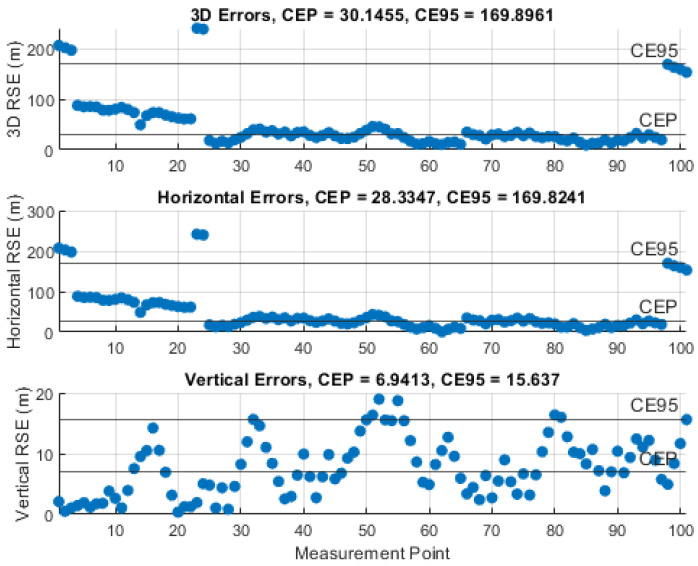
RSE evolution of the IPF solution with a measurement error SD, $\sigma_{\theta ,\varphi }$, of 5°.

The performance of the IPF was demonstrated by monitoring the RSE and computing the circular error probable (CEP), i.e., 50% of the RSEs, and the circular error 95% (CE95), i.e., 95% of the RSEs, using 101 measurements and certain knowledge about the UAS position. The results of the representative simulations, with the investigated measurement noise levels, $\sigma_{\theta ,\varphi }$, are illustrated in Figure 10, Figure 11, Figure 12, Figure 13 and Figure 14. We can see that the CEP approaches the RMSE results at the corresponding measurement noise levels in Table 1. CEP is less accurate by a maximum of about 11 m. Some spikes in Figure 13 and Figure 14 confirm the heavy dependence of the IPF solution on the accuracy of measurements.

All simulation codes were implemented in MATLAB R2020a and were executed on Intel Core i7-8565U central processing unit (CPU) at 1.8 GHz with 24 GB of random-access memory (RAM). The estimated run time of the IPF algorithm (with 1000 particles) to obtain a single emitter location estimate was on the order of 0.3 ms. The estimated batch-processing times of the TSCF, SSCF, and PF (with 5000 particles) solutions using 101 measurement points took about 0.1, 0.1, and 20 ms, respectively.

## 6. Conclusions

ES sensors are passive and relatively small and cheap compared to radar sensors. ES sensors’ silent presence and ease of operation make them hard to detect, avoid, or destroy by adversaries [44]. Accurate passive hostile emitter location is an important issue in ES. It improves situational awareness by allowing the development of EOB and enables evaluation of threat immediacy to reduce response time to threats, develop targeting data, and hand off of threat location information to other friendly platforms.

The signal processing and positioning algorithms using DF, i.e., azimuth and elevation, angle measurements are less complicated than those required when using TDoA and FDoA measurements [45]. Moreover, using DF angles allows a single platform to operate independently to passively detect, identify, and locate threat signal sources from the first emission, e.g., a single pulse, which is a short duration (for example, one-second) emission. Unique antenna array designs and effective DF-based geolocation algorithms allow low-cost, small- and mid-size UASs to cover large geographic areas over extended periods to provide tactical ES information in military missions and to find lost hikers in civilian rescue operations.

This study provided a proof of concept for passive 3D geolocation of stationary emitters from low-cost aerial platforms using as few resources as possible, i.e., only azimuth and elevation measurements, with minimal computational complexity and hardware requirements. The proposed solutions deserve further research efforts to expand the results to other geometries and parameter settings.

Monte-Carlo simulation results have demonstrated the viability of the proposed solutions. It was shown that the PF is a viable approach to 3D geolocation. The IPF can obtain emitter location estimates based on single pulses, eliminating the need for multiple-pulse measurements or multiple platforms. The PF and IPF achieve a good compromise between performance and computational requirements. However, definitive conclusions should not be drawn after a limited set of simulations in a single study. Therefore, a wider scope of simulations and real-world experiments in many independent studies are still required to sufficiently investigate the full capacity of the proposed approaches.

Further investigation and characterization of the proposed algorithms, their application to other geolocation approaches and sensor data fusion problems, and the utilization of digital maps and more geographic information to provide reliable instantaneous geolocation capability are interesting future research directions. The proposed PF, especially, can be modified to assess its performance with other measurement parameters [46,47] in radar surveillance applications [48,49] and multistatic localization scenarios [50].

If emitter detection and data association issues are successfully tackled, the proposed solutions can be straightforwardly extended with minimal modifications to geolocate a vast number of emitters spread over wide areas. Further issues to be addressed include, for example, multipath effects, UAS trajectory, and maneuver selection to improve geolocation performance. Generally, there is no single emitter location approach that will provide high accuracy under all circumstances. Hybrid approaches should be considered to increase performance and to reduce the limitations of the single approaches.

## Figures and Tables

**Table 1:** RMSE results of the batch-processing solutions with certain UAS positions and using 101 measurement points.

Algorithm		$\sigma_{\theta ,\varphi }\;(\text{°})$					Space
		1	2	3	4	5	
**RMSE (m)**	TSCF	13.25	16.16	19.71	25.35	32.78	
	SSCF	12.92	15.32	20.83	28.20	42.37	3D
	PF	14.25	15.49	17.10	20.57	24.46	
	TSCF	13.06	15.56	18.67	23.99	31.22	
	SSCF	12.73	14.64	19.61	26.58	40.12	Horizontal
	PF	14.13	15.28	16.76	20.26	24.19	
	TSCF	2.22	4.35	6.32	8.19	9.98	
	SSCF	2.23	4.52	7.04	9.42	13.62	Vertical
	PF	1.83	2.57	3.38	3.57	3.63	

**Table 2:** RMSE results of the batch-processing solutions with certain UAS positions and using 51 measurement points.

Algorithm		$\sigma_{\theta ,\varphi }\;(\text{°})$					Space
		1	2	3	4	5	
**RMSE (m)**	TSCF	27.06	29.77	35.51	37.86	47.87	
	SSCF	26.74	27.40	32.85	35.03	47.37	3D
	PF	26.52	28.03	29.38	33.55	42.22	
	TSCF	26.80	29.11	34.22	36.36	45.12	
	SSCF	26.48	26.73	31.41	33.20	44.21	Horizontal
	PF	26.47	27.87	29.10	33.33	41.93	
	TSCF	3.74	6.21	9.50	10.54	16.01	
	SSCF	3.76	6.04	9.61	11.18	17.02	Vertical
	PF	1.75	3.02	4.07	3.83	4.94	

**Table 3:** RMSE results of the batch-processing solutions with certain UAS positions and using 11 measurement points.

Algorithm		$\sigma_{\theta ,\varphi }\;(\text{°})$					Space
		1	2	3	4	5	
**RMSE (m)**	TSCF	173.52	176.03	188.37	209.14	210.99	
	SSCF	173.64	170.56	176.69	185.59	181.00	3D
	PF	126.66	130.74	132.55	147.69	160.95	
	TSCF	170.58	172.91	183.79	203.49	203.66	
	SSCF	170.66	167.47	172.48	180.49	174.15	Horizontal
	PF	126.50	130.61	132.37	147.52	160.79	
	TSCF	31.83	33.02	41.28	48.28	55.13	
	SSCF	32.03	32.29	38.36	43.18	49.33	Vertical
	PF	6.51	5.97	6.78	7.14	7.13	

## Data Availability

Not applicable.

## Abbreviations

2D	Two-dimensional
3D	Three-dimensional
AoA	Angle of arrival
ASE	Aircraft survivability equipment
CE95	Circular error 95%
CEP	Circular error probable
CF	Closed-form
CTLS	Constrained total least squares
DD	Differential Doppler
DF	Direction finding
DoA	Direction of arrival
DPD	Discrete probability density
EA	Electronic attack
ECCM	Electronic counter-countermeasures
ECM	Electronic countermeasures
ELINT	Electronic intelligence
EM	Electromagnetic
EMI	Electromagnetic interference
EOB	Electronic order of battle
EP	Electronic protection
ERP	Equivalent radiated power
ES	Electronic support
ESM	Electronic support measures
EW	Electronic warfare
FDoA	Frequency difference of arrival
GDoP	Geometric dilution of precision
GNSS	Global navigation satellite system
IPF	Instantaneous particle filter
ISR	Intelligence, surveillance, and reconnaissance
LLS	Linear least squares
LoB	Line of bearing
LS	Least squares
ML	Maximum likelihood
NLoS	Non-line of sight
NLS	Nonlinear least squares
PCE	Polynomial chaos expansion
PDF	Probability density function
PDoA	Phase/Power difference of arrival
PF	Particle filter
PoI	Probability of intercept
RF	Radiofrequency
RMS	Root mean square
RMSE	Root mean square error
RSE	Root square error
RSS	Received signal strength
RSSD	Received signal strength difference
RWR	Radar warning receiver
SD	Standard deviation
SNR	Signal-to-noise ratio
SSCF	Single-step closed-form
SSL	Single-site location
SWaP	Size, weight, and power
TDoA	Time difference of arrival
TLS	Total least squares
ToA	Time of arrival
TSCF	Two-step closed-form
TTP	tactics, techniques, procedures
UAS	Unmanned aircraft system
WLS	Weighted least squares
WTAE	Weighted trimmed average estimate

## References

[1] Bamberger R.J., Moore J.G., Goonasekeram R.P., Scheidt D.H. (2013). Autonomous Geolocation of RF Emitters Using Small, Unmanned Platforms. Johns Hopkins APL Tech. Dig..

[2] Joint Publication (JP) 3-13-1: Joint Doctrine for Electronic Warfare; Joint Chiefs of Staff: Washington, DC, USA, 2012. https://fas.org/irp/doddir/dod/jp3-13-1.pdf.

[3] Inkol R. Electronic Warfare. Defence Research and Development Canada, Ottawa, ON, Canada, DRDC-OTTAWA-SL-2008-019, 29 May 2008. https://cradpdf.drdc-rddc.gc.ca/PDFS/unc306/p806524_A1b.pdf.

[4] Wang S., Inkol R., Jackson B.R. Relationship between the maximum likelihood emitter location estimators based on received signal strength (RSS) and received signal strength difference (RSSD). Proceedings of the 2012 26th Biennial Symposium on Communications (QBSC).

[5] Qu X., Xie L., Tan W. (2017). Iterative constrained weighted least squares source localization using TDOA and FDOA measurements. IEEE Trans. Signal. Process..

[6] Zhang R., Liu J., Du X., Li B., Guizani M. (2018). AOA-Based Three-Dimensional Multi-Target Localization in Industrial WSNs for LOS Conditions. Sensors.

[7] Kang S., Kim T., Chung W. (2020). Hybrid RSS/AOA Localization using Approximated Weighted Least Square in Wireless Sensor Networks. Sensors.

[8] Le A.T., Tran L.C., Huang X., Ritz C., Dutkiewicz E., Phung S.L., Bouzerdoum A., Franklin D. (2020). Unbalanced Hybrid AOA/RSSI Localization for Simplified Wireless Sensor Networks. Sensors.

[9] O’Donoughue N. (2020). Emitter Detection and Geolocation for Electronic Warfare.

[10] Lee K., Kim S., You K. (2021). Iterative Regression Based Hybrid Localization for Wireless Sensor Networks. Sensors.

[11] Avionics Department (2013). Electronic Warfare and Radar Systems Engineering Handbook.

[12] Houry M., Troussel A. Why Instant 3D Geolocation Will Become Standard in Airborne ISR. Avantix SAS, 18 March 2020. https://avantix.net/why-instant-3d-geolocation-will-become-standard-in-airborne-isr/.

[13] Stansfield R.G. (1947). Statistical theory of D.F. fixing. J. IEE.

[14] Foy W. (1976). Position-Location Solutions by Taylor-Series Estimation. IEEE Trans. Aerosp. Electron. Syst..

[15] Torrieri D. (1984). Statistical Theory of Passive Location Systems. IEEE Trans. Aerosp. Electron. Syst..

[16] Poirot J.L., Mcwilliams G.V. (1974). Application of linear statistical models to radar location techniques. IEEE Trans. Aerosp. Electron. Syst..

[17] Gavish M., Weiss A.J. (1992). Performance analysis of bearing-only target location algorithms. IEEE Trans. Aerosp. Electron. Syst..

[18] Brown R.M. (1981). Emitter Location Using Bearing Measurements from a Moving Platform.

[19] Pages-Zamora A., Vidal J., Brooks D.H. Closed-form solution for positioning based on angle of arrival measurements. Proceedings of the 13th IEEE International Symposium on Personal, Indoor and Mobile Radio Communications.

[20] Cheung K.W., So H.C., Ma W.-K., Chan Y.T. (2006). A constrained least squares approach to mobile positioning: Algorithms and optimality. EURASIP J. Adv. Signal. Process..

[21] Rao K.D., Reddy D. (1994). A new method for finding electromagnetic emitter location. IEEE Trans. Aerosp. Electron. Syst..

[22] Sun G., Xu X., Yang K. Angle-of-Arrival based Constrained Total Least-Squares Location Algorithm. Proceedings of the 2012 National Conference on Information Technology and Computer Science.

[23] Elsaesser D. The Discrete Probability Density Method for Emitter Geolocation. Proceedings of the 2006 Canadian Conference on Electrical and Computer Engineering.

[24] Vorst T.V., Eeckhaute M.V., Benlarbi-Delai A., Sarrazin J., Quitin F., Horlin F., Doncker P. Angle-of-Arrival based localization using polynomial chaos expansions. Proceedings of the Workshop on Dependable Wireless Communications and Localization for the IoT.

[25] Paradowski L. Unconventional algorithm for emitter position location in three-dimensional space using data from two-dimensional direction finding. Proceedings of the 1994 IEEE National Aerospace and Electronics Conference (NAECON).

[26] Paik J.W., Lee J.-H. (2019). Noniterative Three-Dimensional Location Estimation Using Azimuth and Elevation Measurements at Multiple Locations. J. Sens..

[27] Haystead J. (2018). Tactical HF SIGINT/Geolocation Systems Tackle Elusive Challenges. J. Electron. Def..

[28] Fu S.J., Vian J.L., Grose D.L. (1988). Determination of ground emitter location. IEEE Aerosp. Electron. Syst. Mag..

[29] (2000). Electronic Warfare Fundamentals.

[30] Adamy D. (2003). Introduction to Electronic Warfare Modeling and Simulation.

[31] Poisel R. (2012). Electronic Warfare Target Location Methods.

[32] Tuncer T., Friedlander B. (2009). Classical and Modern Direction-of-Arrival Estimation.

[33] Graham A. (2011). Communications, Radar and Electronic Warfare.

[34] Adamy D. (2008). EW 103: Tactical Battlefield Communications Electronic Warfare.

[35] Adamy D. (2015). EW 104: EW Against a New Generation of Threats.

[36] Sherman S., Barton D. (2011). Monopulse Principles and Techniques.

[37] Guo F., Fan Y., Zhou Y., Xhou C., Li Q. (2014). Space Electronic Reconnaissance: Localization Theories and Methods.

[38] Huie L., Fowler M. Emitter location in the presence of information injection. Proceedings of the 2010 44th Annual Conference on Information Sciences and Systems (CISS).

[39] Figueiras J., Frattasi S. (2010). Mobile Positioning and Tracking.

[40] Gordon N.J., Salmond D.J., Smith A.F.M. (1993). Novel approach to nonlinear/non-Gaussian Bayesian state estimation. IEE Proc. F Radar Signal. Process..

[41] Khalaf-Allah M. (2020). Particle Filtering for Three-Dimensional TDoA-Based Positioning Using Four Anchor Nodes. Sensors.

[42] Khalaf-Allah M. (2008). Nonparametric Bayesian Filtering for Location Estimation, Position Tracking, and Global Localization of Mobile Terminals in Outdoor Wireless Environments. EURASIP J. Adv. Signal. Process..

[43] Magers M. (2016). Geolocation of RF Emitters Using a Low-Cost UAV-Based Approach. Master’s Thesis.

[44] Smestad T., Ohra H., Knapskog A. ESM-Sensors for Tactical Information in Air Defence Systems. Proceedings of the RTO SCI Symposium on System Concepts for Integrated Air Defense of Multinational Mobile Crisis Reaction Forces.

[45] Grabbe M.T., Hamschin B.M. (2013). Geo-location using direction finding angles. Johns Hopkins APL Tech. Dig..

[46] Beck A., Stoica P., Li J. (2008). Exact and Approximate Solutions of Source Localization Problems. IEEE Trans. Signal. Process..

[47] Shen J., Molisch A., Salmi J. (2012). Accurate Passive Location Estimation Using TOA Measurements. IEEE Trans. Wirel. Commun..

[48] Aubry A., Braca P., Demaio A., Marino A. (2021). 2D PBR Localization Complying with Constraints Forced by Active Radar Measurements. IEEE Trans. Aerosp. Electron. Syst..

[49] Aubry A., Braca P., De Maio A., Marino A. (2021). Enhanced Target Localization with Deployable Multiplatform Radar Nodes Based on Non-Convex Constrained Least Squares Optimization. arXiv.

[50] Zhang Y., Ho K.C. (2019). Multistatic Localization in the Absence of Transmitter Position. IEEE Trans. Signal. Process..

